# Targeted Assessment of the *EGFR* Status as Reflex Testing in Treatment-Naive Non-Squamous Cell Lung Carcinoma Patients: A Single Laboratory Experience (LPCE, Nice, France)

**DOI:** 10.3390/cancers12040955

**Published:** 2020-04-13

**Authors:** Sandra Lassalle, Véronique Hofman, Simon Heeke, Jonathan Benzaquen, Elodie Long, Michel Poudenx, Elisabeth Lantéri, Jacques Boutros, Virginie Tanga, Katia Zahaf, Salomé Lalvée, Virginie Lespinet, Olivier Bordone, Jean-Marc Félix, Christelle Bonnetaud, Charles Marquette, Marius Ilie, Paul Hofman

**Affiliations:** 1 Laboratory of Clinical and Experimental Pathology, Pasteur Hospital, University Côte d’Azur, 30 avenue de la voie romaine, 06000 Nice, France; 2 Hospital-Related Biobank (BB-0033-00025), Pasteur Hospital, University Côte d’Azur, 30 avenue de la voie romaine, 06000 Nice, France; 3 FHU OncoAge, Pasteur Hospital, University Côte d’Azur, 30 avenue de la voie romaine, 06000 Nice, France; 4 IRCAN Inserm 1081/CNRS 7284, Antoine Lacassagne Center, avenue de valombrose, 06002 Nice, France; 5 Department of Pneumology, Pasteur Hospital, University Côte d’Azur, 30 avenue de la voie romaine, 06000 Nice, France

**Keywords:** lung cancer, *EGFR*, targeted sequencing, next generation sequencing

## Abstract

Background: Assessment of actionable *EGFR* mutations is mandatory for treatment-naïve advanced or metastatic non-squamous lung carcinoma (NSLC), but the results need to be obtained in less than 10 working days. For rapid *EGFR* testing, an *EGFR*-specific polymerase chain reaction (PCR) assay is an alternative and simple approach compared to next generation sequencing (NGS). Here, we describe how a rapid *EGFR*-specific PCR assay can be implemented in a single laboratory center (LPCE, Nice, France) as reflex testing in treatment-naïve NSLC. Methods: A total of 901 biopsies from NSLC with more than 10% of tumor cells were prospectively and consecutively evaluated for *EGFR* mutation status between November 2017 and December 2019 using the Idylla system (Biocartis NV, Mechelen, Belgium). NGS was performed for nonsmokers with NSLC wild type for *EGFR*, *ALK*, *ROS1*, and *BRAF* and with less than 50% PD-L1 positive cells using the Hotspot panel (Thermo Fisher Scientific, Waltham, MA, USA). Results: Results were obtained from 889/901 (97%) biopsies with detection of *EGFR* mutations in 114/889 (13%) cases using the Idylla system. Among the 562 *EGFR* wild type tumors identified with Idylla, NGS detected one actionable and one nonactionable *EGFR* mutation. Conclusions: Rapid and targeted assessment of *EGFR* mutations in treatment-naïve NSLC can be implemented in routine clinical practice. However, it is mandatory to integrate this approach into a molecular algorithm that allows evaluation of potentially actionable genomic alterations other than *EGFR* mutations.

## 1. Introduction

Around 15–40% of patients with non-squamous lung carcinoma (NSLC) harbor specific mutations in the *EGFR* gene. The frequency is higher in females, Asians, and nonsmokers [1,2,3]. It is noteworthy that patients with *EGFR*-mutated NSLC and a history of smoking show response to *EGFR* tyrosine kinase inhibitor (TKI) treatment, but their outcomes are usually not as good as never-smoking patients [4]. Moreover, both never-smokers and smokers have improvement in progression-free survival (PFS) with TKI therapy compared to chemotherapy. Treatment with *EGFR* TKI resulted in 36% greater benefit to never-smokers than current or former smokers [5]. The detection of actionable *EGFR* mutations allows administration of TKI-targeted therapies for late stage NSLC patients, which is the current standard-of-care [6]. Consequently, the molecular assessment of *EGFR* is mandatory for correct treatment selection in NSLC [6].

According to international guidelines, *EGFR* status must be obtained in less than 10 working days to allow rapid initiation of therapy [7]. In addition, evaluation of other genomic alterations (rearrangement of *ALK*, *ROS1*, and *BRAF* V600 mutations) is also mandatory in treatment-naïve late stage NSLC [6,7]. Since immunotherapy can be proposed alone or in combination with first-line chemotherapy in patients whose tumors are wild type for *EGFR*, *ALK*, *ROS1*, and *BRAF*, it is obligatory to obtain the PD-L1 status of tumor cells [7]. However, some actionable targets can be present in other genes (notably *MET*, *RET*, *NTRK*, and *HER2*), allowing inclusion into clinical trials of patients with *EGFR*, *ALK*, *ROS1*, and *BRAF* wild-type tumors with less than 50% PD-L1-positive tumor cells. In this case, assessment and validation by a board to evaluate the molecular status of tumors and treatment at an expert lung cancer center are highly recommended.

The detection of *EGFR* mutations is commonly performed using a specific polymerase chain reaction (PCR) assay or next generation sequencing (NGS) technology. NGS is currently the method of choice for lung tumor genotyping in many academic hospital centers, particularly at baseline. Different panels of genes can be used for NGS to allow physicians to obtain not only the mandatory *EGFR*, *ALK*, *ROS1*, and *BRAF* status, but also other genomic alterations that allow some patients to be included into clinical trials. In this context, numerous laboratories implemented NGS in routine clinical care to answer clinicians’ requests. Thus, interest in using a single *EGFR*-specific PCR assay decreased substantially over the last few years.

Consequently, we evaluated if the assessment of the *EGFR* status in NSLC biopsies using an *EGFR*-specific PCR assay still has a place in the daily practice of an academic hospital center, and how this single gene testing approach could be integrated into lung cancer patient care.

## 2. Results

A total of 901/1368 (66%) biopsies had more than a 10% tumor cell content, allowing for the assessment of *EGFR* mutations using the Idylla assay. A total of 889/901 (97%) biopsies tested using the Idylla assay yielded a successful result. *EGFR* mutations were detected in 114/889 (13%) cases using the Idylla system (Table 1).

Among the *EGFR* wild-type tumors, subsequent NGS identified two *EGFR* mutations that were not present in the Idylla *EGFR* test panel, as well as some genomic alterations of interest, notably three cases with a *HER2* mutation (Table 2). However, none of these five patients received additional targeted therapy. Two patients with *EGFR* mutations had early-stage lung cancer and did not receive adjuvant treatment. Two patients with *HER2* mutations died before a therapeutic decision was made. One patient with an *HER2* mutation received chemotherapy after consideration of the high tumor burden by a medical board, but died three weeks later.

**Figure 1:**
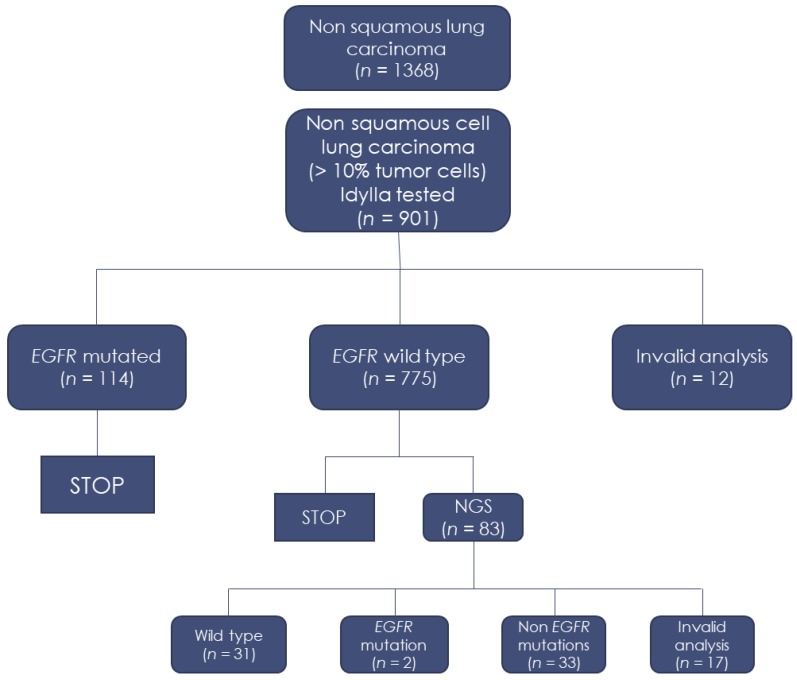
Flowchart of the study.

The immunohistochemistry (IHC) results identified 31/889 (3.5%) cases with strong ALK staining, 14/889 (1.4%) cases with ROS1-positive staining (all confirmed by *ROS1* fluorescence in-situ hybridization [FISH]) and 22/889 (2.5%) cases with positive BRAFV600E staining. A total of 265/889 (30%) biopsies showed a PD-L1 IHC with more than 50% positive tumor cells. The flowchart of this study and the main results are shown in Figure 1.

The turnaround times (TAT) were two days (range of one to three days) and eight days (range of four to sixteen days) for the Idylla and NGS workflows, respectively. The TATs did not take into consideration the time for transport from the clinical department to the clinical pathological laboratory or for the technical procedures and histological diagnosis.

## 3. Discussion

In comparison to an NGS approach, the use of a specific PCR assay for *EGFR* mutation assessment in NSLC is questionable for an academic hospital center [8,9], as NGS allows the simultaneous evaluation of numerous genomic alterations across several genes. However, the NGS approach shows some limitations in routine clinical practice for *EGFR* mutation detection, including that the TAT is usually longer than the TAT associated with the specific PCR assay, the need for specially qualified personnel, the cost of equipment and different reagents, the absence of reimbursement of these tests, which is not guaranteed in all countries, and limited availability in some laboratories. The present work showed the usefulness of an *EGFR* specific PCR assay using the Idylla system in a single hospital center due to its specificity, sensitivity, and rapidity, since it is very easy to use in a pathology laboratory. Moreover, this assay was easily integrated into the clinical practice of pathologists. In our experience, this approach allowed the laboratory to limit the number of systematic NGS demands made by the physicians for patient care, notably at baseline for treatment-naïve NSLC patients, without prolonging the assessment time compared to NGS.

**Figure 2:**
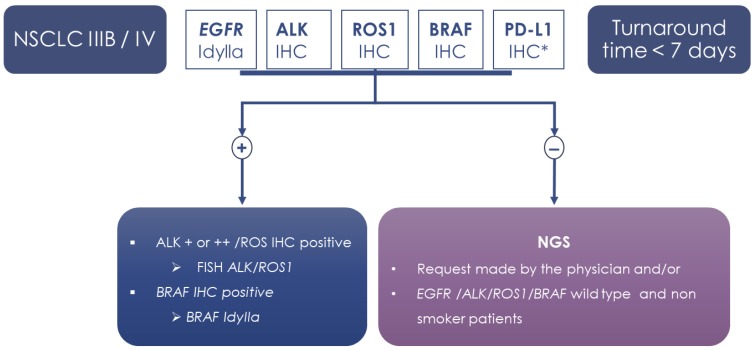
Flowchart of the study. * PD-L1: NSCLC all stages.

In our institution (LPCE, Nice, France), the daily practice for the evaluation of NSLC biopsies includes molecular (*EGFR*) and ALK, ROS1, BRAF, and PD-L1 IHC as reflex testing (Figure 2). These predictive biomarkers are systematically analyzed after histological diagnosis, even if the pTNM staging is only partially known or unknown at this time. Admittedly, for a certain percentage of patients with early stage tumors (12% of patients in the present study), these tests should be avoided in theory. Consequently, for patients with stage I–IIIA NSLC, the presence of detected genomic alterations in *EGFR*, *ALK*, *ROS1*, and *BRAF* would not result in adjuvant targeted therapy. However, the reflex testing described above allowed time to be saved in therapeutic decision-making for a large majority of late-stage lung cancer patients.

The number of single-gene molecular assays, notably for the evaluation of *EGFR* status, decreased rapidly in molecular pathology platforms developed in academic hospital centers. For example, 28 French National Cancer Institute genetic platforms were rapidly equipped with NGS, replacing previously used single-gene tests [10]. Due to the rapid emergence of new targeted therapies for different genomic alterations for lung cancer patients and the growing complexity of the molecular landscape of lung cancer, rapid development of NGS approaches using different gene panels was necessary. Moreover, NGS screens many mutations to select patients for inclusion into clinical trials, notably into phase I trials [11]. In this setting, physicians can obtain a large profile of genomic alterations and prepare a therapeutic strategy based on the molecular analysis. While this is an ideal situation in centers with access to several clinical trials, comprehensive molecular screening at baseline may be questioned in routine clinical practice, particularly in hospital centers without phase I clinical trial units. According to the international European guidelines, besides PD-L1 IHC, the mandatory molecular tests for untreated late-stage NSLC patients concern only a few genes, namely, *EGFR*, *ALK*, *ROS1*, and *BRAF*. The genetic status of these four genes can be assessed rapidly using specific molecular and immunohistochemical assays, significantly reducing the TAT in comparison to NGS. In first-line treatment, a patient receives an *EGFR*, *ALK*, *ROS1* or *BRAF* genomic alteration targeted therapy or immunotherapy (with or without chemotherapy) for tumors without druggable alterations in the respective genes. Other second-line treatments may be proposed upon tumor progression for cases with genomic alterations in *RET, MET, NTRK, HER2,* or other genes, but the patient is also included into a clinical trial most of the time, at least in France. Obtainment of an additional tumor biopsy and NGS analysis of the sample are usually recommended, since some new alterations can be present after tumor progression. Second-line immunotherapy may also be proposed if PD-L1 IHC is positive in more than 1% of the tumor cells present in tissue sections or in certain countries depending on the expression of PD-L1 in the tumor cells. In this context, NGS practice at baseline for broad screening of molecular events could presently be judged as excessive or even as a non-useful, costly approach. According to the quantity and quality of the extracted nucleic acids, the sensitivity of molecular testing can be variable. This is certainly an important issue, since the biological material obtained for lung cancer morphological and molecular biology diagnosis is limited, frequently containing only a few percent of tumor cells. Furthermore, the detection of *ALK* and *ROS1* rearrangements using NGS can be challenging in comparison with IHC and FISH approaches, notably due to the quality of the RNA on formalin-fixed tissue and the quantity required for multiplex approaches. The TAT to obtain the results of predictive biomarkers in thoracic oncology is critical for optimal treatment selection for late-stage lung cancer patients. Thus, protracted *EGFR* status assessment may force the patient to undergo ineffective and/or toxic treatment, such as chemotherapy and/or immunotherapy [12]. In this regard, a few medical centers developed rapid specific tests targeting *EGFR* as well as simultaneous NGS testing using the same biopsy [13]. TAT is dependent on the delay between the biopsy procedure and arrival at the clinical and molecular laboratories. Different steps need to be taken into account, such as transmission of the biopsy sample to the clinical pathology laboratory, pre-analytical and analytical times, post-analytical time for histological diagnosis and immunohistochemistry, transmission of the formalin-fixed paraffin embedded (FFPE) tumor to the molecular pathology laboratory, nucleic acid extraction and sequencing time, molecular analysis and quality control, and finally diffusion of the report. Reducing time in regard to *EGFR* mutation assessment is high priority, and ultra-rapid methods of detection can be mandatory for urgent treatment of patients with *EGFR* TKIs [12]. In this regard good sensitivity and specificity of the rapid detection of *EGFR* mutations in tissue and cytological samples using the Idylla system were reported by ourselves and others [14,15,16,17,18]. More importantly, this is a system that delivers *EGFR* testing results within a few hours, when NGS usually takes up to one or two weeks.

Considering the continuing increase in the incidence of lung cancer and the development of a number of new therapeutic molecules, the cost and interest of the different tests for predictive biomarker detection must be taken into consideration due to the low level of reimbursement rates, as well as the financial sustainability of molecular laboratories in certain countries, including France. Thus, cost is variable according to the country, the laboratory organization, the politics of the institution, the annual number of different tests performed per laboratory, and the molecular tests (e.g., type of NGS panels, probes, nature of the clones used for IHC). NGS testing, notably when using some comprehensive cancer panels, is globally more expensive than specific targeted assays [19]. The different *EGFR* mutations detected using the specific Idylla *EGFR* assay and the commercially available NGS panels are quite similar, even if some rare discrepancies may be present. More than 96% of the different *EGFR* mutations can be detected using both the Idylla *EGFR* test and the NGS hotspot panel. Certain rare mutations detected in the latter panel are absent in the Idylla *EGFR* test panel. However, the impact of this discrepancy on patient care is very low, as efficient and approved therapies are missing for those mutations [20,21]. Thus, these very rare mutations were not reported to be actionable for the different TKIs [22,23,24]. It is important to be aware of the possibility of obtaining a false-negative *EGFR* mutation using the Idylla device, however, only one discrepancy on druggable alterations was observed, highlighting the reliability of the Idylla device [25]. The false-negative results may be due to the sensitivity of the technique for *EGFR* detection, in particular in the case of a very low allele frequency, but also in the case of a low percentage of tumor cells with the *EGFR* mutation in the tissue section before DNA extraction. Importantly, no false-positive calls were observed in our study in the retrospective cohort of 80 samples.

## 4. Materials and Methods

A retrospective study of 80 NSLC biopsies was first performed as a training set. These tumors were comparatively assessed for *EGFR* mutations using the *EGFR* assay with the Idylla device (Biocartis) and the Hotspot V2 panel with an ion S5 sequencer (Thermo Fisher, Waltham, MA, USA), as described previously [26,27]. Briefly, the Idylla method was carried out using the same tissue blocks as those used for the NGS method; a 10 μm thick FFPE tissue section was loaded directly into the cartridge using sterile forceps and was then placed in the Idylla system, allowing integrated DNA extraction and mutational hotspot analysis. In case of a discrepancy between the results using these two methods (i.e., an *EGFR* mutation detected with the NGS approach rather than the Idylla approach) an orthogonal method using dPCR (Stilla, Villejuif, France) was performed as described [28]. Briefly, the purity of the DNA was assessed by absorbance using a Nanodrop spectrophotometer (ThermoFischer, Waltham, MA, USA) prior to quantification by a fluorescent method using the Qubit Fluorimeter (ThermoFischer, Waltham, MA, USA). Oligonucleotide primers and hydrolysis probes were synthesized by Eurogentec (Liège, Belgium) in RP-HPLC-grade. MixPCR reactions were carried out using the PerfeCta Multiplex qPCRToughMix (Quanta Biosciences, Gaithersburg, MD, USA). To allow adequate imaging of all droplets for software analysis, FITC (Saint Louis, MO, USA) was added to each reaction to reach a final concentration of 40 nM. The Naica Geode was programmed to perform the sample partitioning step, followed by the PCR thermal cycling program: 95 °C for 10 min, followed by 45 cycles of 95 °C for 10 s and 60 °C for 15 s. Total droplet enumeration and droplet quality control, enabled by the detection of the reference dye FITC in the Blue channel, was performed using Crystal Reader software.

Following technical evaluation, a prospective analysis was done from November 2017 to December 2019 on a consecutive series of 1368 patients hospitalized in Nice University Hospital (Nice, France), who had NSLC diagnosed by bronchial or transthoracic biopsies. In parallel, ALK (D5F3, Ventana, Tucson, AZ, USA), ROS1 IHC (D4D6, Cell Signaling), BRAFV600E (VE1, Ventana), and PD-L1 (Dako, Carpenteria, CA, USA) immunohistochemistry was performed according to previous studies [29,30,31]. Briefly, specimens were sectioned at a thickness of 3 μm and stained on positively charged glass slides stored at 4 °C within one day after sectioning. Deparaffinization and antigen retrieval was performed with CCI (prediluted; pH 8.0) antigen retrieval solution (ref. No. 950–124, Ventana Medical Systems, Inc.), performed using the the Ventana BenchMark ULTRA automated slide stainer. Specimens were incubated with the above-cited primary antibodies, followed by visualization using the OptiView DAB IHC Detection Kit (Ventana) and OptiView Amplification (Ventana). Finally, the specimens were counterstained with hematoxylin II and bluing reagent (Ventana) and coverslipped. *ALK* FISH was performed in the case of weak or moderate ALK IHC staining, as described previously [32]. *ROS1* FISH was performed for positive ROS1 IHC as described [29] (Figure 2).

*EGFR* mutation analysis was performed on the fully automated Idylla platform following the manufacturer’s protocol, as previously described [16,26]. Briefly, one tissue section of 10 μm was used for each biopsy and placed directly in the appropriate Idylla *EGFR* mutation cartridge. The main epidemiological and pathological data of the patients included in this study are reported in Table 3. NGS was performed in (i) *EGFR* wild-type tumors after Idylla analysis of non-smokers and (ii) on specific request made by the physician (e.g., in the case of a young patient, and/or rapid tumor progression, and/or for potential inclusion in a clinical trial), representing 83/889 (9%) cases in total. The different *EGFR* mutations detected according to the technology used were compared. Moreover, the TATs of Idylla and NGS, from the registry of the FFPE tissue block in the pathology molecular department to emission of the report, were compared.

## 5. Conclusions

In conclusion, certain teams, including ours, limit the use of NGS at baseline for certain patients and integrate the practice of a large sequencing approach using a specific algorithm for predictive biomarker testing [33]. The algorithm proposed to physicians for patient care could be modified and should be adapted to the circuit of the samples, the different therapeutic strategies available, the requests of the physicians, and the financial model of the molecular pathology laboratory. NGS using large panels is promising for the detection of new therapeutic targets, even though recent studies showed that there is currently little benefit to real-life patients [11]. Similarly, in our study, which included a limited number of patients, the NGS results did not allow the inclusion of a patient into a clinical trial. However, it is reasonable to argue that therapeutic molecules targeting additional genes other than *EGFR*, *ALK*, *ROS1*, and *BRAF* would obtain FDA and EMA approval in the near-future for use in front-line care of late-stage non-squamous lung carcinoma. This is the case for drugs targeting *NTRK* fusion that were recently approved by the FDA and EMA. In this context, the assessment of *EGFR* status using a single-gene assay may be very useful in the near-future, even for treatment-naïve patients. Moreover, the cost of NGS should soon come down and become more competitive for single-gene testing [34]. Concomitant mutational evaluation after NGS analysis, particularly when looking for genomic alterations associated with *EGFR* mutation, may change TKI indications for these patients in the future, thus encouraging systematical molecular assessment by NGS [2,35,36].

## Figures and Tables

**Table 1:** *EGFR* mutations detected using the Idylla system.

Idylla System	Number (%)
Invalid	12 (1.3%)
No mutation detected	775 (86%)
Del exon 19	51 (5.6%)
Del exon 19 + T790M	3 (0.3%)
L858R	34 (3.9%)
L858R + T790M	1 (0.1%)
L861Q	10 (1.1%)
G719A/C/S	5 (0.6%)
G719A/C/S + S768I	4 (0.5%)
S768I	3 (0.3%)
Ins exon 20	3 (0.3%)

**Table 2:** *EGFR* mutations not present in the Idylla panel and *HER2* mutations detected using the hotspot next generation sequencing (NGS) panel.

Idylla Panel	Hotspot NGS Panel	Genes
Wild type	p.L747_S752 > Q, c.2239_2256 > CAA	*EGFR* Exon 19
Wild type	p.P772_H773dup, c.2314_2319dup	*EGFR* Exon 20
Not applicable	c.2325_2326insTCCGTGATGGCT; p.Ala775_Gly77linsSerValMetAla	*HER2*
Not applicable	c.2325_2330delins18; p.Gly776_Val777delinsTyrValMetAlaGlyGly	*HER2*
Not applicable	c.2585C > T; p.Thr862Ile	*HER2*

**Table 3:** Main epidemiological and pathological data.

Age (Years)	Mean (Range)	66 (36–98)
Sex	F	363 (40.3%)
	M	538 (59.7%)
Smoking history	Smokers	707 (78.5%)
	Non-smokers	95 (10.5%)
	Unknown	99 (11%)
Stage	I	13 (1.4%)
	II	25 (2.8%)
	III	115 (12.8%)
	IV	465 (51.6%)
	Unknown (at the time of the histological diagnosis)	283 (31.4%)
Samples	Surgical lymph node biopsy	52 (5.8%)
	Surgical pleural biopsy	48 (5.3%)
	Core needle biopsy	202 (22.5%)
	Bronchial biopsy	599 (66.4%)
Histological type	Lung adenocarcinoma	795 (88.2%)
	NSCLC NOS	91 (10.1%)
	Large cell carcinoma	15 (1.7%)
Tumor cellularity	Mean (Range)	45% (15–95%)
Type of pathological material	Formalin fixed paraffin embedded	872 (97%)
	Fresh tissue	29 (3%)
